# Days Alive and out of Hospital at 30 Days After Curative Gastrectomy for Gastric Adenocarcinoma: Complication-Severity Gradient and Readmission Burden

**DOI:** 10.3390/jcm15176811

**Published:** 2026-09-02

**Authors:** Adem Ozcan, Gizem Gunes, Ali Bal, Abdulkadir Unsal

**Affiliations:** Department of Surgical Oncology, Ankara Bilkent City Hospital, Ankara 06800, Türkiye

**Keywords:** gastric adenocarcinoma, gastrectomy, days alive and out of hospital, DAOH30, postoperative complications, Clavien–Dindo classification, readmission, Charlson comorbidity index

## Abstract

**Background/Objectives:** Days alive and out of the hospital at 30 days (DAOH30) integrates survival, index hospitalization, and readmission. We characterized DAOH30 after gastrectomy, assessed its complication-severity gradient and readmission burden, and explored its association with an index-cancer-excluded Charlson Comorbidity Index (CCI). **Methods:** This single-center retrospective analysis included adults undergoing R0 total gastrectomy or subtotal distal gastrectomy for non-metastatic gastric adenocarcinoma between January 2020 and April 2026. DAOH30 was derived from the index postoperative length of stay, readmission days, and 30-day mortality. Rank-based tests and median quantile regression were used. **Results:** Among 123 patients, median DAOH30 was 21 days (interquartile range, 18–22; range, 4–26). No 30-day deaths occurred; eight patients (6.5%) were readmitted. Median DAOH30 decreased from 22 days without complications to 21 days after Clavien–Dindo grade I–II complications and 17 days after grade ≥ III complications (Kruskal–Wallis *p* < 0.001; one-sided Jonckheere–Terpstra *p* < 0.001; two-sided permutation sensitivity *p* < 0.001). After adjustment, grade ≥ III complications were associated with 4.00 fewer median DAOH30 days (bootstrap 95% confidence interval, −7.31 to −2.25; model-based *p* < 0.001). Readmissions contributed 73 additional inpatient days, with a median decrement of 9.5 days per readmitted patient. No statistically significant independent association was detected between the index-cancer-excluded CCI and DAOH30 (adjusted median difference, −0.17 days per point; bootstrap 95% confidence interval, −1.34 to 0.40; model-based *p* = 0.640). **Conclusions:** DAOH30 summarized early hospital burden, but in this no-mortality, low-readmission cohort it was largely determined by index length of stay. Multicenter validation with patient-reported anchoring is warranted.

## 1. Introduction

Gastric cancer continues to impose a substantial global disease burden despite advances in prevention, molecular classification, systemic treatment, and multidisciplinary care [1]. For patients with resectable gastric adenocarcinoma, curative-intent gastrectomy with appropriate lymphadenectomy remains a central component of treatment in contemporary international guidelines [2,3]. Nevertheless, gastrectomy is a physiologically demanding intervention, and international benchmark data demonstrate clinically meaningful variation in postoperative morbidity, hospital stay, and recovery even among experienced centers [4]. Technical success and oncological adequacy are therefore necessary but insufficient descriptors of the early burden experienced by patients after surgery.

Postoperative recovery has traditionally been reported through separate outcomes such as mortality, complication grade, length of stay, reoperation, and readmission. Each endpoint is clinically relevant, but each captures only one dimension of the postoperative course. Mortality is definitive but uncommon after elective surgery; complication classifications describe therapeutic severity but not the duration of its consequences; length of stay is influenced by institutional discharge pathways; and readmission does not account for the burden of the index hospitalization. Consequently, apparently favorable performance according to one isolated outcome may coexist with substantial cumulative hospital exposure. A recovery measure capable of integrating these interrelated dimensions may provide a more coherent representation of the early postoperative experience.

Days alive and out of the hospital within 30 days after surgery (DAOH30) addresses this limitation by combining survival, the index hospitalization, and subsequent acute-care admissions within a fixed observation window. Prolonged index admission and readmission progressively reduce DAOH30, while death within the observation period is assigned the lowest possible value. The Standardised Endpoints in Perioperative Medicine initiative recommended DAOH30 as a life-impact endpoint for perioperative research [5]. Large prospective and population-based studies subsequently demonstrated that time-at-home measures are feasible, clinically interpretable, sensitive to patient and procedural risk, and strongly associated with postoperative complications and longer-term outcomes [6,7]. A recent scoping review further supported their use as flexible perioperative outcomes with potential patient relevance, while emphasizing the need for explicit definitions, consistent observation windows, and transparent handling of readmissions and death [8].

Time-at-home outcomes may be particularly informative in surgical oncology, where the consequences of treatment extend beyond the occurrence of an isolated complication. Population-based cancer surgery data suggest that time spent outside healthcare institutions may reflect functional independence and the cumulative burden of institutional care [9]. Gastrectomy presents a distinctive recovery profile because changes in gastrointestinal continuity, nutritional intake, metabolic reserve, and postoperative inflammatory stress may lead to prolonged admission or clinically important events after discharge. Readmission following curative gastrectomy has been associated with total gastrectomy, pre-existing medical disease, postoperative complications, nutritional difficulties, and infectious morbidity [10,11]. DAOH30 therefore has the potential to quantify a dimension of postoperative burden that the index length of stay alone cannot fully capture: the additional hospital exposure generated by early readmission. It should not, however, be interpreted as intrinsically superior to length of stay; rather, it extends that measure by incorporating survival and hospital use after discharge.

Baseline comorbidity may influence postoperative recovery, but disease-weighted comorbidity and realized postoperative burden are conceptually distinct. The Charlson Comorbidity Index (CCI) provides a reproducible summary of chronic disease burden and has been associated with short- and long-term outcomes after gastrectomy [12,13]. Postoperative morbidity and readmission remain clinically consequential after gastric cancer surgery [14,15], yet whether greater Charlson burden translates into fewer days alive outside the hospital remains uncertain. We therefore treated the association between the index-cancer-excluded CCI and DAOH30 as an exploratory secondary question.

Accordingly, the primary objective of this study was to characterize DAOH30 after R0 total gastrectomy or subtotal distal gastrectomy for gastric adenocarcinoma. The key secondary objectives were to evaluate the gradient in DAOH30 across increasing Clavien–Dindo complication severity and to quantify the additional hospital exposure attributable to 30-day readmission. The exploratory objective was to examine the association between preoperative comorbidity burden, measured using the index-cancer-excluded CCI, and DAOH30. Based on the clinical ordering of the Clavien–Dindo categories, we expected DAOH30 to decrease progressively with increasing complication severity.

## 2. Materials and Methods

### 2.1. Study Design, Setting, and Ethical Oversight

This retrospective, single-center, observational cohort study was conducted at the Surgical Oncology Clinic of Ankara Bilkent City Hospital, Ankara, Türkiye. A study-specific Surgical Oncology database was assembled from routine clinical, operative, inpatient, and follow-up records of adults who underwent gastrectomy for gastric adenocarcinoma between January 2020 and April 2026.

The retrospective protocol governing abstraction of routine-care demographic, comorbidity, operative, postoperative, hospitalization, readmission, and mortality data was approved by the Ankara Bilkent City Hospital Medical Research Scientific and Ethical Evaluation Committee (Approval No. TABED 1-26-2582; Approval Date: 6 May 2026). The present study was conducted as a post hoc secondary analysis in which DAOH30 and the index-cancer-excluded CCI were derived from variables collected under the approved protocol. All index operations included in the analysis had been completed before ethics approval as part of routine clinical care. Study-specific eligibility assessment, structured data abstraction, source-document verification, de-identification, and statistical analysis were initiated after approval.

No patient was prospectively recruited, and no aspect of diagnosis, surgery, perioperative management, or postoperative follow-up was modified for research purposes. No additional patient contact, intervention, laboratory test, imaging examination, or research-specific follow-up visit was undertaken. The study was based exclusively on de-identified data generated during routine clinical care and was conducted in accordance with the Declaration of Helsinki. Reporting followed the Strengthening the Reporting of Observational Studies in Epidemiology recommendations for cohort studies [16].

The analytic database was finalized after 30-day outcome ascertainment had been completed for the last eligible operation. Direct identifiers were removed before analysis, and each patient was assigned a study-specific code.

### 2.2. Patient Identification and Eligibility

Patients were identified from the Surgical Oncology clinical, operative, inpatient, and outpatient follow-up records. Supporting pre-anesthesia assessments, operative reports, final pathology reports, discharge summaries, hospital admission records, and relevant specialist documentation were reviewed when required for verification.

Patients entered the study-specific dataset if they were aged ≥18 years; had histopathologically confirmed non-metastatic gastric adenocarcinoma; underwent elective R0 curative-intent gastrectomy under the clinical responsibility of the Surgical Oncology Clinic; and had chronologically valid information for the operation date, index postoperative length of stay, 30-day vital status, and 30-day readmission status, together with sufficient source-documented preoperative comorbidity information to calculate the index-cancer-excluded CCI. For patients who were readmitted, admission and discharge dates were also required.

Patients undergoing emergency surgery, palliative or otherwise non-curative resection, non-R0 resection, surgery for metastatic disease, or surgery in the presence of a synchronous malignancy were excluded. Application of these non-procedural clinical and data-completeness criteria yielded a study-specific dataset of 132 patients. Nine patients who underwent proximal gastrectomy were subsequently excluded according to the procedure-specific eligibility criterion, leaving 123 patients who underwent total gastrectomy or subtotal distal gastrectomy in the primary analytic cohort.

**Figure 1:**
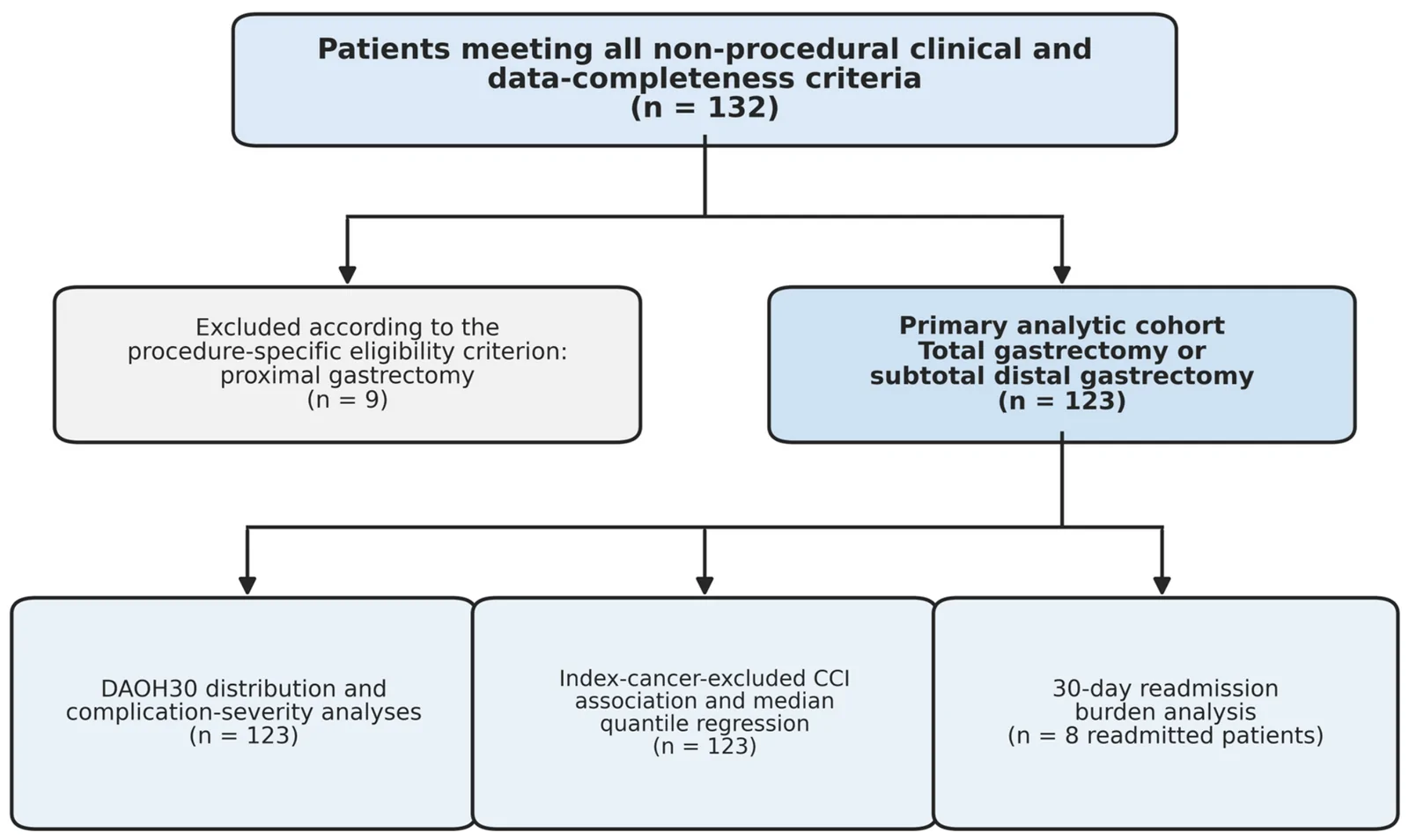
Derivation of the study-specific analytic cohort and analysis sets. The study-specific dataset comprised 132 patients meeting all non-procedural clinical and data-completeness criteria. Nine patients undergoing proximal gastrectomy were excluded according to the procedure-specific eligibility criterion, leaving 123 patients who underwent total gastrectomy or subtotal distal gastrectomy. All 123 patients were included in the DAOH30 distribution, complication-severity, and index-cancer-excluded CCI analyses; readmission-related burden was characterized among the eight readmitted patients. DAOH30, days alive and out of the hospital within 30 days; CCI, Charlson Comorbidity Index.

Eligibility was determined without reference to DAOH30, complication severity, readmission duration, or any subsequent postoperative outcome. The derivation of the study-specific analytic cohort is shown in Figure 1.

### 2.3. Relationship to a Related Analysis

A related analysis from the same Surgical Oncology practice addresses perioperative CALLY dynamics, quantitative nodal tumor burden, textbook surgical outcome, recurrence, and survival. The present study examined distinct outcomes and did not repeat those analyses. The related manuscript is disclosed to the Editorial Office in the accompanying cover letter.

### 2.4. Data Collection and Quality Assurance

Demographic, preoperative, operative, and postoperative variables were abstracted retrospectively from Surgical Oncology records and supporting source documentation.

Variables retained for the present analysis included:

- Age and sex;
- American Society of Anesthesiologists physical-status classification;
- Eastern Cooperative Oncology Group performance status;
- Pre-existing comorbid conditions;
- Receipt of neoadjuvant treatment;
- Type of gastrectomy;
- Surgical approach;
- Operation date;
- Index postoperative length of stay;
- Postoperative complication type and severity;
- Anastomotic leakage;
- Reoperation within 30 days;
- All-cause inpatient readmission within 30 days of surgery;
- Readmission admission and discharge dates;
- 30-day all-cause mortality.

All patient-level dates used in calculating DAOH30 were verified against Surgical Oncology documentation and the corresponding hospitalization records. Thirty-day readmission status was considered ascertainable only when the available hospitalization records and Surgical Oncology follow-up documentation permitted confirmation of whether an inpatient admission had occurred during the postoperative observation window. Admissions outside the institutional network could be identified only when documented during Surgical Oncology follow-up; otherwise, they could not be independently verified. For readmitted patients, the operation date, index length of stay, readmission date, and readmission discharge date were cross-checked to establish a chronologically valid sequence.

A hospitalization beginning before completion of the index admission was not classified as a separate readmission. Continuous inpatient care or interfacility transfer without an intervening period outside the hospital was treated as a single hospitalization. Overlapping hospital days were not counted more than once.

Internal-consistency controls were performed for operation–discharge chronology, operation–readmission chronology, readmission admission–discharge chronology, index length of stay, 30-day mortality, Clavien–Dindo grade, major-complication status, anastomotic leakage, and reoperation. No missing DAOH30 component or index-cancer-excluded CCI component was imputed. All analytic variables were derived from source-verified clinical records.

### 2.5. Assessment of Preoperative Comorbidity Burden

Pre-existing comorbid conditions were determined from information documented before the index gastrectomy. Source documents included Surgical Oncology clinical records, pre-anesthesia assessments, previous discharge summaries, specialist evaluations, active diagnosis lists, and other relevant routine-care documentation.

A condition was scored only when it represented an established preoperative diagnosis. Transient perioperative abnormalities, postoperative complications, and diagnoses first documented during or after the index hospitalization were not classified as baseline comorbidities.

Charlson components were weighted according to the original framework [17]. Conditions assigned one point comprised myocardial infarction, congestive heart failure, peripheral vascular disease, cerebrovascular disease, dementia, chronic pulmonary disease, connective-tissue disease, peptic-ulcer disease, mild liver disease, and diabetes without chronic complications. Diabetes with chronic complications, hemiplegia or paraplegia, moderate-to-severe renal disease, leukemia, and lymphoma received two points; moderate-to-severe liver disease received three points; and metastatic solid tumor or acquired immunodeficiency syndrome received six points. Mutually exclusive severity categories, including uncomplicated versus complicated diabetes and mild versus moderate-to-severe liver disease, were not scored simultaneously.

The primary comorbidity exposure was a modified CCI that omitted the uniform weight assigned to the index non-metastatic gastric malignancy; this measure is hereafter termed the index-cancer-excluded CCI. All other original Charlson condition weights were retained. Because the index malignancy was present in all participants, removal of this constant did not alter patient ranking or the slope of continuous within-cohort associations. Chronological age was not incorporated into this score and was modeled separately. The index-cancer-excluded CCI was analyzed continuously per one-point increase. Categories of 0, 1, 2, and ≥3 were used for descriptive and sensitivity analyses only and should not be interpreted as externally validated risk strata.

An age-adjusted index-cancer-excluded CCI was calculated by adding the established age weights to the index-cancer-excluded comorbidity score [18]. When this score was used in a sensitivity model, chronological age was not entered separately, thereby avoiding duplicate adjustment for age.

The prevalence of individual Charlson components is presented in Supplementary Table S1.

### 2.6. Surgical and Perioperative Care

The extent of gastrectomy was selected according to tumor location, oncological resectability, and multidisciplinary clinical assessment. Total gastrectomies and subtotal distal gastrectomies were included in the primary cohort; proximal gastrectomies were excluded in accordance with the study-specific eligibility criteria.

At the study center, postoperative intensive care admission after gastrectomy was predominantly a planned monitoring pathway. Accordingly, intensive care admission itself was not treated as an adverse event, a known-groups criterion, or an explanatory variable for DAOH30. Planned intensive care monitoring without organ dysfunction was not classified as a postoperative complication or a Clavien–Dindo grade IV event. Unexpected escalation beyond the planned pathway was classified according to the underlying clinical event and the corresponding Clavien–Dindo criteria. Throughout the study period, the principal institutional gastrectomy pathway—including routine postoperative intensive care monitoring, nutritional advancement, and clinical discharge criteria—remained broadly stable, and no formal pathway change was implemented that was expected to systematically alter postoperative length of stay.

### 2.7. Postoperative Complications and Readmission

Postoperative events occurring within 30 days of the index operation were identified through review of source-verified progress notes, operative and interventional reports, intensive-care documentation, and discharge summaries and were classified according to the Clavien–Dindo system [19,20]. Clavien–Dindo grades were assigned retrospectively by two surgical investigators, and classification disagreements were resolved by consensus with a third surgical investigator. For each patient, the highest Clavien–Dindo grade occurring within 30 days was retained for analysis. Grade III or higher events were defined as major postoperative complications.

For the present analysis, the postoperative course was categorized as:

- No postoperative complication;
- Clavien–Dindo grade I–II complication;
- Clavien–Dindo grade ≥ III complication.

Anastomotic leakage and reoperation within 30 days were retained as separate clinically relevant outcomes. Routine planned postoperative intensive care admission was not included in the definition of morbidity.

A qualifying readmission was defined as an all-cause inpatient admission occurring after discharge from the index hospitalization and within 30 days of the operation date. Emergency-department attendance or outpatient evaluation without inpatient admission was not counted as a readmission.

For any admission extending beyond postoperative day 30, only the portion overlapping the 30-day postoperative observation window contributed to DAOH30. If more than one qualifying hospitalization occurred, all non-overlapping inpatient days within the observation window were summed.

### 2.8. Definition and Calculation of DAOH30

The primary endpoint of the present secondary analysis was days alive and out of hospital within 30 days after surgery (DAOH30). DAOH30 was evaluated as a post hoc derived endpoint using routinely recorded index length of stay, 30-day readmission, and 30-day mortality data.

The operation date was used as the index date. The 30-day observation window extended from the operation date, defined as postoperative day 0, up to but not including the calendar date 30 days later. Hospital exposure was counted using occupied hospital nights; consequently, the discharge date was considered a day outside the hospital.

DAOH30 was calculated as:

DAOH30 = 30 − index postoperative length of stay − readmission days within the 30-day window

Index postoperative length of stay was defined as the calendar-day difference between the operation date and index discharge date, consistent with the Surgical Oncology length-of-stay variable. Only index-hospital and readmission days overlapping the 30-day observation window were subtracted. A patient remaining continuously hospitalized through the end of the observation window would therefore have a DAOH30 value of 0.

Readmission days were calculated as the calendar-day difference between the readmission date and the corresponding discharge date. Hospital days occurring after the end of the observation period did not reduce DAOH30. Patients who died from any cause within 30 days of surgery were assigned a DAOH30 value of 0, irrespective of any period previously spent outside the hospital [6,7].

For patients who survived 30 days without readmission:

DAOH30 = 30 − index postoperative length of stay

Among patients alive at postoperative day 30, the readmission-related decrement was calculated as:

Readmission decrement = (30 − index postoperative length of stay) − DAOH30

Among 30-day survivors, this quantity was algebraically equivalent to the number of readmission days falling within the observation window. No readmission-related decrement was calculated for patients who died within 30 days because DAOH30 was assigned a value of 0. DAOH30 was retained as a continuous outcome. No data-driven DAOH30 threshold was generated for the primary analysis.

### 2.9. Study Outcomes

The primary objective was to characterize the distribution of DAOH30 after R0 total gastrectomy or subtotal distal gastrectomy.

The key secondary objectives were:

1. To evaluate known-groups discrimination across increasing Clavien–Dindo complication severity;
2. To quantify the decrement in days outside the hospital attributable to 30-day readmission.

The exploratory objectives were:

1. To evaluate the association between preoperative index-cancer-excluded CCI and DAOH30;
2. To describe DAOH30 according to major-complication status, anastomotic leakage, 30-day reoperation, and gastrectomy type.

Readmission status was not analyzed as an independent explanatory or validation variable because readmission days are a direct mathematical component of DAOH30. Its contribution was instead quantified through the absolute readmission-related decrement.

The association between DAOH30 and index length of stay was considered descriptive. Because index length of stay is also a mathematical component of DAOH30, this relationship was not interpreted as evidence that DAOH30 was superior to length of stay.

### 2.10. Statistical Analysis

All 123 patients fulfilling the study-specific eligibility criteria were included in the analyses. No formal a priori sample-size calculation was performed because the study included the complete available study-specific cohort.

Continuous variables were assessed using histograms, Q–Q plots, and the Shapiro–Wilk test. Approximately normally distributed variables were summarized as mean ± standard deviation; non-normally distributed variables were summarized as median and interquartile range. Categorical variables were reported as numbers and percentages.

DAOH30 was treated as a bounded continuous outcome and was summarized using the mean and standard deviation, median and interquartile range, minimum and maximum, and a histogram.

DAOH30 was not dichotomized according to its sample median, receiver operating characteristic analysis, or the Youden index in the primary analysis.

#### 2.10.1. Known-Group Discrimination

DAOH30 was compared across the ordered groups of no postoperative complication, Clavien–Dindo grade I–II complication, and Clavien–Dindo grade ≥ III complication.

The overall comparison was performed using the Kruskal–Wallis test. Based on the clinical ordering of the Clavien–Dindo categories, a directional decrease in DAOH30 with increasing complication severity was evaluated using a one-sided Jonckheere–Terpstra test. The directional hypothesis was not formally prespecified in the original study protocol. Therefore, a two-sided permutation sensitivity analysis was additionally performed by comparing the absolute deviation of the observed Jonckheere–Terpstra statistic from its permutation null expectation with the corresponding permutation distribution. Both permutation *p* values were based on 50,000 label permutations and were calculated using the +1 Monte Carlo correction. When the overall comparison was significant, pairwise comparisons were performed using Dunn’s test with Holm adjustment.

Group differences were supplemented by nonparametric bootstrap estimates of median differences with 95% confidence intervals based on 100,000 resamples.

Supportive two-group comparisons were performed according to major-complication status, 30-day reoperation, anastomotic leakage, and gastrectomy type using Mann–Whitney U tests. Median differences were accompanied by 100,000-resample nonparametric bootstrap 95% confidence intervals, and distributional effect sizes were expressed using Cliff’s δ. These analyses were exploratory, and their *p* values were not adjusted for multiplicity. Routine planned intensive care admission was not included in these analyses.

#### 2.10.2. Readmission-Related Hospital Burden

For readmitted patients, the contribution of readmission to early hospital exposure was summarized as:

- Total additional inpatient days within the 30-day window;
- Median additional inpatient days per readmitted patient;
- Interquartile range;
- Minimum and maximum.

Because readmission days are algebraically embedded in DAOH30, no causal interpretation was assigned to the association between readmission status and DAOH30.

#### 2.10.3. Exploratory Association Between Index-Cancer-Excluded CCI and DAOH30

The unadjusted association between continuous index-cancer-excluded CCI and DAOH30 was evaluated using Spearman’s rank correlation. Differences in DAOH30 across index-cancer-excluded CCI categories were evaluated using the Kruskal–Wallis test, and the ordinal association between index-cancer-excluded CCI category and DAOH30 was assessed using Kendall’s τb.

The adjusted association between index-cancer-excluded CCI and DAOH30 was evaluated using median quantile regression at the 0.50 quantile. Regression coefficients were reported as adjusted differences in median DAOH30 days.

The preoperative Charlson association model included:

- Index-cancer-excluded CCI, per one-point increase;
- Age, per 10-year increase;
- Sex;
- ASA class III–IV versus I–II;
- Total versus subtotal gastrectomy;
- Receipt of neoadjuvant treatment;
- Calendar year of surgery.

To evaluate adjusted known-groups discrimination, a separate median quantile regression model included complication-severity group, with no postoperative complication as the reference category, together with index-cancer-excluded CCI, age, sex, ASA class, and gastrectomy type. Covariates were selected before outcome modeling on the basis of clinical relevance and to limit model complexity. ECOG performance status and ASA classification were not entered simultaneously in the preoperative Charlson association model. For both the preoperative Charlson association model and the adjusted complication-severity model, 95% percentile-bootstrap confidence intervals were estimated using 5000 nonparametric case-resampling samples. Model-based *p* values were reported separately, whereas bootstrap confidence intervals constituted the primary inferential measure.

Postoperative complications, index length of stay, and readmission were not included in the preoperative Charlson association model because they occurred after surgery and represented mediators or direct mathematical components of DAOH30.

Multicollinearity was assessed using variance inflation factors. Model fit was summarized using the Koenker–Machado pseudo-R^2^. No stepwise selection, automated predictor screening, nomogram construction, or machine-learning model was performed. The regression analysis was explanatory rather than a prediction-model development exercise.

Sensitivity analyses included:

1. Replacing the index-cancer-excluded CCI and separate age variable with the age-adjusted index-cancer-excluded CCI;
2. Modeling the index-cancer-excluded CCI categorically as 0, 1, 2, or ≥3;
3. Replacing ASA classification with ECOG performance status;
4. Restricting the analysis to patients undergoing open gastrectomy.

Confidence intervals for the sensitivity models were model-based, as specified in Supplementary Table S4.

No missing values were imputed. Analyses were performed using complete data for the variables included in each model. Pairwise known-groups comparisons were adjusted using the Holm procedure; other secondary and sensitivity analyses were interpreted principally according to effect estimates and 95% confidence intervals.

All inferential tests were two-sided except the primary directional Jonckheere–Terpstra test; the ordered trend was also evaluated using a two-sided permutation sensitivity analysis as described above. A *p* value < 0.05 was considered statistically significant.

Fixed random-number seeds were specified before the permutation and bootstrap procedures to ensure computational reproducibility. Analyses were performed using IBM SPSS Statistics for Windows, version 31.0 (IBM Corp., Armonk, NY, USA), and Python version 3.13.5 with NumPy 2.3.5, SciPy 1.17.0, statsmodels 0.14.6, and scikit-learn 1.8.0. Figures were generated using Matplotlib 3.10.8.

### 2.11. Use of Generative Artificial Intelligence

During manuscript preparation, ChatGPT (GPT-5.6 Pro, OpenAI, San Francisco, CA, USA) was used for language editing, structural organization, construction of a data-validation template, and assistance with selected analytic code and visualizations. The tool was not used as a source of final patient-level clinical data, and no unverified AI-generated value was retained in the analytic dataset. All final patient-level variables were independently verified against the source Surgical Oncology records before analysis. All AI-assisted text, code, numerical outputs, references, figures, and interpretations were independently reviewed by the authors, who take full responsibility for the final work.

## 3. Results

### 3.1. Cohort Assembly and Clinical Profile

The study-specific dataset comprised 132 patients who met all non-procedural clinical and data-completeness criteria. Nine patients undergoing proximal gastrectomy were excluded according to the procedure-specific eligibility criterion, leaving 123 patients in the primary analytic cohort: 57 underwent subtotal distal gastrectomy (46.3%) and 66 underwent total gastrectomy (53.7%) (Figure 1). Complete DAOH30, index-cancer-excluded CCI, and covariate data were available for all 123 patients included in the analyses.

The median age was 67 years (interquartile range [IQR], 57–75), and 73 patients (59.3%) were male. American Society of Anesthesiologists class III–IV was present in 67 patients (54.5%), Eastern Cooperative Oncology Group performance status was at least 1 in 91 (74.0%), and 22 (17.9%) received neoadjuvant treatment. Most procedures were performed using an open approach (*n* = 118, 95.9%).

The median index-cancer-excluded CCI was 1 (IQR, 0–1). Fifty patients (40.7%) had an index-cancer-excluded CCI of 0, 45 (36.6%) had a score of 1, 17 (13.8%) had a score of 2, and 11 (8.9%) had a score of ≥3. The median age-adjusted index-cancer-excluded CCI was 3 (IQR, 2–4). The complete clinical and early postoperative characteristics are presented in Table 1, and the prevalence of individual Charlson components is provided in Supplementary Table S1.

### 3.2. Distribution and Component Structure of DAOH30

**Figure 2:**
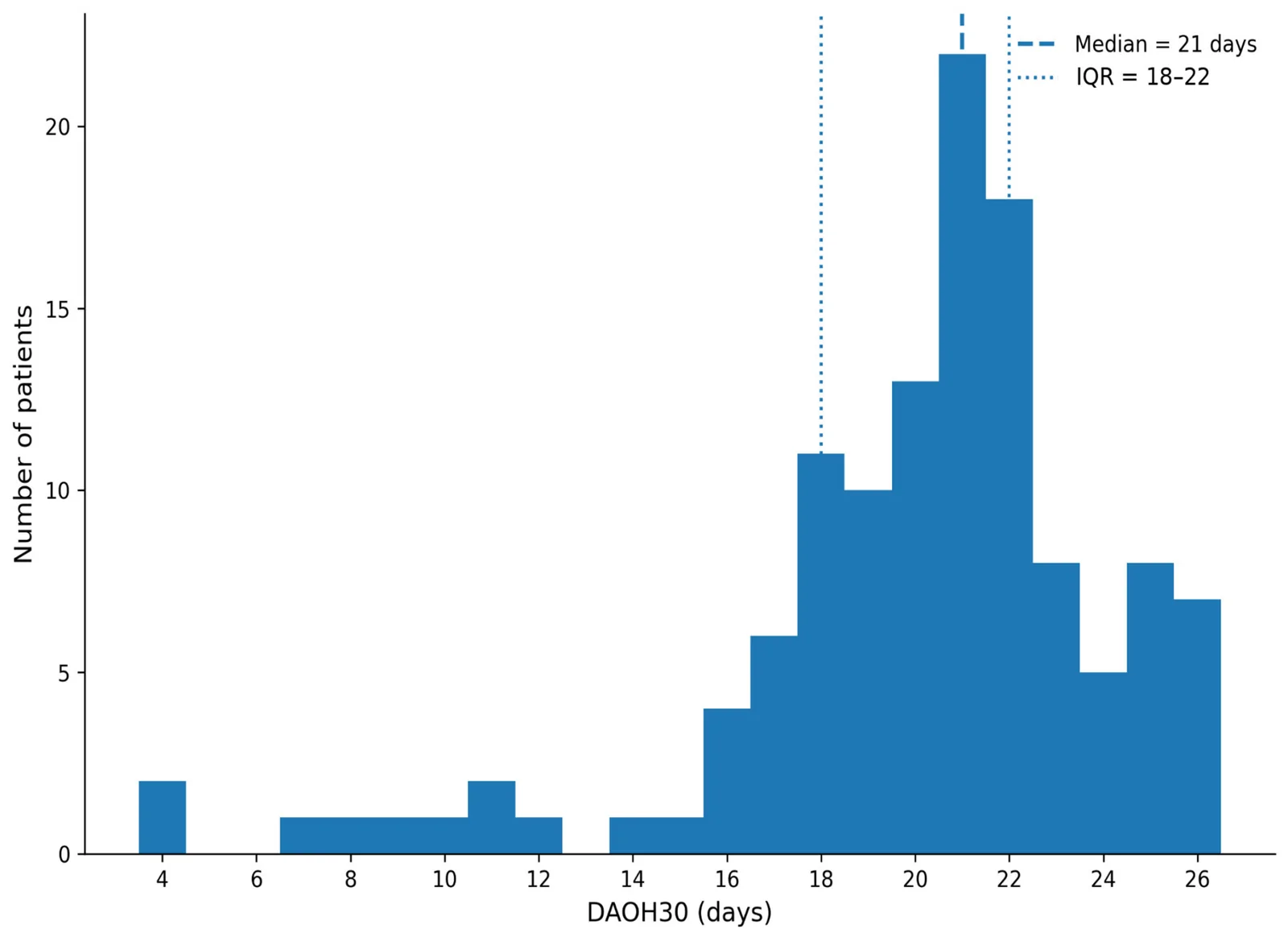
Distribution of DAOH30 after R0 total gastrectomy or subtotal distal gastrectomy (*n* = 123). The dashed vertical line denotes the median of 21 days, and the dotted lines denote the first and third quartiles of 18 and 22 days, respectively. DAOH30, days alive and out of the hospital within 30 days.

DAOH30 was non-normally distributed (Shapiro–Wilk *p* < 0.001) and showed a left-skewed distribution (skewness, −1.54). The mean DAOH30 was 20.0 ± 4.2 days, and the median was 21 days (IQR, 18–22; range, 4–26) (Figure 2). No patient had a floor value of 0 or the theoretical ceiling value of 30. Because no death occurred within 30 days, variation in DAOH30 reflected differences in index hospitalization and early readmission burden.

The median index postoperative length of stay was 9 days (IQR, 8–11). As expected from the construction of the endpoint, DAOH30 correlated strongly and inversely with index length of stay (Spearman ρ = −0.940; *p* < 0.001). Because 115 of 123 patients (93.5%) survived 30 days without readmission, DAOH30 was algebraically equivalent to 30 minus the index length of stay in most of the cohort; the incremental information beyond index length of stay arose from the eight readmission episodes.

### 3.3. Known-Group Discrimination Across Postoperative Complication Severity

**Figure 3:**
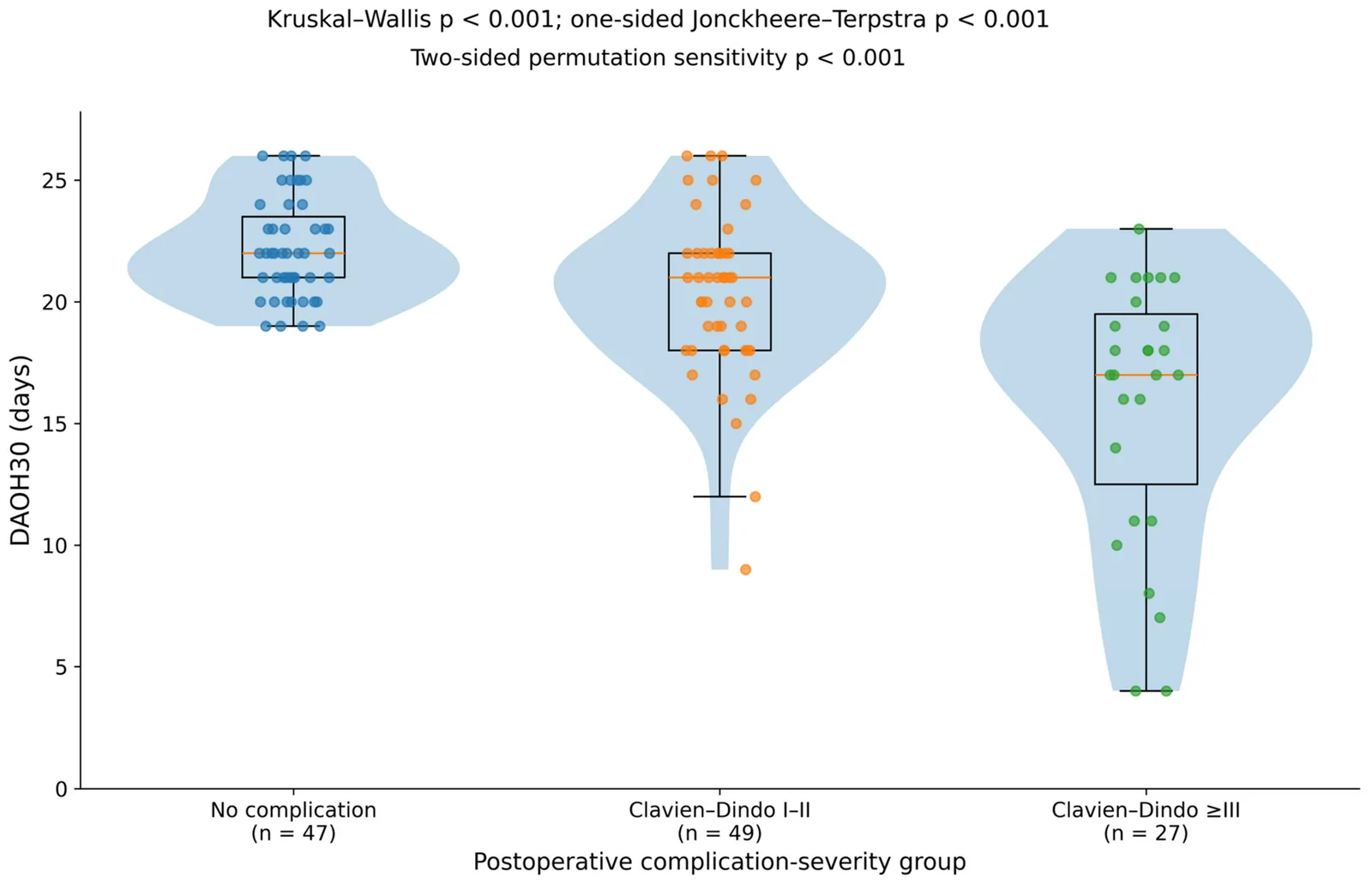
DAOH30 according to postoperative complication severity. Violin widths represent distribution density, embedded box plots show medians and interquartile ranges, and points represent individual patients. Orange horizontal lines indicate group medians. DAOH30 decreased progressively across ordered complication-severity groups (Kruskal–Wallis *p* < 0.001; one-sided Jonckheere–Terpstra *p* < 0.001; two-sided permutation sensitivity *p* < 0.001). DAOH30, days alive and out of the hospital within 30 days.

DAOH30 decreased progressively with increasing Clavien–Dindo severity. The median DAOH30 was 22 days (IQR, 21–23.5) in patients without a documented complication, 21 days (IQR, 18–22) in those with grade I–II complications, and 17 days (IQR, 12.5–19.5) in those with grade ≥ III complications (Kruskal–Wallis H = 36.40; *p* < 0.001; one-sided Jonckheere–Terpstra *p* < 0.001; two-sided permutation sensitivity *p* < 0.001) (Table 2; Figure 3).

The DAOH30 rank distribution was lower in patients with grade I–II complications than in those without complications (Holm-adjusted Dunn *p* = 0.005; Cliff’s δ = −0.35), whereas the estimated median difference was −1 day (bootstrap 95% CI, −3 to 0). Patients with grade ≥ III complications had lower DAOH30 than those without complications (median difference, −5 days; bootstrap 95% CI, −6 to −3; Holm-adjusted Dunn *p* < 0.001; Cliff’s δ = −0.81) and those with grade I–II complications (median difference, −4 days; bootstrap 95% CI, −5 to −1; Holm-adjusted Dunn *p* < 0.001; Cliff’s δ = −0.54) (Supplementary Table S2).

In supportive analyses, major complications were associated with a 4-day lower median DAOH30 (bootstrap 95% CI, −6 to −2; Mann–Whitney *p* < 0.001; Cliff’s δ = −0.67). The DAOH30 rank distribution was also lower among patients who underwent reoperation (Mann–Whitney *p* = 0.009; Cliff’s δ = −0.52), whereas the estimated median difference was −3 days (bootstrap 95% CI, −5 to 0). The anastomotic-leak comparison was directionally similar but imprecise because only five leaks occurred, whereas DAOH30 did not differ significantly according to gastrectomy type (Table 2).

### 3.4. Adjusted Complication-Severity Analysis

After adjustment for index-cancer-excluded CCI, age, sex, ASA class, and gastrectomy type, grade ≥ III complications were associated with 4.00 fewer median DAOH30 days compared with patients without postoperative complications (5000-resample bootstrap 95% CI, −7.31 to −2.25; model-based *p* < 0.001) (Table 3B). The corresponding contrast for grade I–II complications was −1.00 day (bootstrap 95% CI, −3.00 to 0.00; model-based *p* = 0.180). The model had a Koenker–Machado pseudo-R^2^ of 0.136.

### 3.5. Readmission-Related Hospital Burden

Eight patients (6.5%) were readmitted within 30 days. Readmission began at a median of postoperative day 14 (IQR, 12–17.5). These episodes contributed 73 additional inpatient days within the 30-day postoperative window, corresponding to a median readmission-related decrement of 9.5 days per readmitted patient (IQR, 7.5–11.3; range, 5–12).

Across the full cohort, readmission increased cumulative 30-day hospital exposure from 1154 index-hospital days to 1227 total hospital days, representing a 6.3% increment beyond the index hospitalization alone. Four readmissions were related to intra-abdominal abscess or sepsis, two to infection or fever, and two to nutritional, dehydration, or other medical causes. Among readmitted patients, the highest recorded Clavien–Dindo grade was II in four patients, IIIa in three, and IIIb in one (Supplementary Table S3).

**Figure 4:**
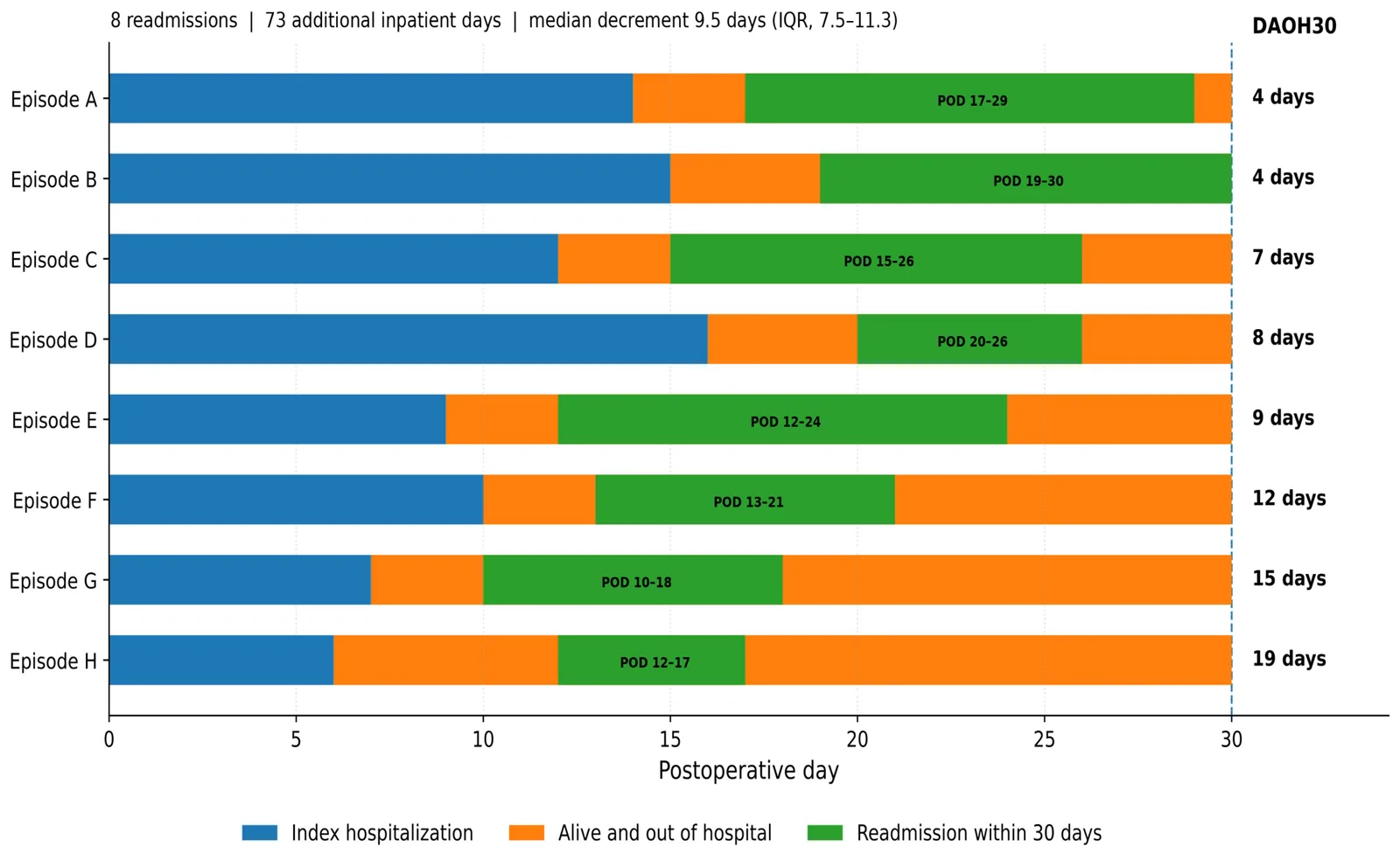
Patient-level temporal decomposition of the first 30 postoperative days among readmitted patients. Each row represents one readmission episode and is ordered by ascending DAOH30. The operation date was defined as postoperative day 0. Index hospitalization, time alive and out of the hospital, and readmission are displayed according to their actual temporal positions within the 30-day postoperative window. Labels within the readmission segments indicate the postoperative days on which readmission began and ended. DAOH30 values are shown on the right. The eight readmissions contributed 73 additional inpatient days, with a median readmission-related decrement of 9.5 days (interquartile range, 7.5–11.3). Only inpatient days overlapping the first 30 postoperative days reduced DAOH30. DAOH30, days alive and out of the hospital within 30 days; POD, postoperative day.

The temporal decomposition of the first 30 postoperative days among readmitted patients is shown in Figure 4. The episodes were ordered by ascending DAOH30, and the readmission intervals were positioned according to their actual postoperative timing. No inferential test was applied to readmission status itself because readmission days are an algebraic component of DAOH30.

### 3.6. Exploratory Association Between Index-Cancer-Excluded CCI and DAOH30

A weak inverse correlation was observed between the index-cancer-excluded CCI and DAOH30; however, no statistically significant association was detected (Spearman ρ = −0.138; *p* = 0.129). No statistically significant difference in DAOH30 was detected across index-cancer-excluded CCI categories (Kruskal–Wallis *p* = 0.391), and no statistically significant ordinal association was detected (Kendall’s τb = −0.108; *p* = 0.136) (Supplementary Table S5). The prevalence of individual Charlson components is presented in Supplementary Table S1.

In the preoperative Charlson association model, the adjusted median difference in DAOH30 per one-point increase in the index-cancer-excluded CCI was −0.17 days (5000-resample bootstrap 95% CI, −1.34 to 0.40; model-based *p* = 0.640); therefore, no statistically significant independent association was detected (Table 3A). The 95% confidence intervals for age, sex, ASA class, gastrectomy type, neoadjuvant treatment, and calendar year of surgery also included zero. The model had a Koenker–Machado pseudo-R^2^ of 0.020, and all variance inflation factors were ≤1.27.

## 4. Discussion

This single-center study provides an initial procedure-specific evaluation of DAOH30 after R0 total gastrectomy or subtotal distal gastrectomy. DAOH30 decreased across increasing complication severity, with grade ≥ III complications associated with four fewer median days after adjustment, and eight readmissions contributed 73 additional inpatient days. However, because no 30-day deaths occurred and 93.5% of patients survived without readmission, DAOH30 was largely determined by the index postoperative length of stay. The findings therefore support DAOH30 as a summary of realized early hospital burden and known-groups discrimination in this cohort, rather than establishing independent prognostic information or incremental clinical value beyond conventional postoperative measures.

The structural relationship with length of stay is central to interpretation. In 115 of 123 patients, DAOH30 was algebraically equivalent to 30 minus the index postoperative length of stay, and its additional information arose only from the eight readmission episodes; the mortality component of the endpoint was not observed. Accordingly, the present study demonstrates feasibility and known-groups discrimination but does not establish superiority, incremental predictive value, or net clinical benefit over length of stay. Larger multicenter cohorts with greater variation in readmission and early mortality are required to evaluate those questions.

DAOH combines survival, index hospitalization, and subsequent acute-care use within a fixed window, providing a clinically interpretable summary of early hospital exposure [5,6,7,8]. Prior cancer-surgery studies, most often using DAOH90, have linked less time at home with complications, impaired performance status, greater healthcare use, and worse subsequent outcomes [21,22,23,24]. Direct numerical comparison remains inappropriate because observation windows, procedures, healthcare settings, and handling of death differ. The 30-day window used here is particularly sensitive to index hospitalization, early complications, and readmission, whereas longer windows additionally capture rehabilitation, delayed nutritional problems, and transitions to oncological treatment.

The clearest evidence of known-groups discrimination was the monotonic gradient in DAOH30 across increasing complication severity. Patients without a complication had a median DAOH30 of 22 days, compared with 21 days after grade I–II complications and 17 days after grade ≥ III complications. The magnitude and ordering of these differences are clinically coherent with the Clavien–Dindo framework, in which increasing grades reflect progressively more intensive treatment requirements [19,20]. The marked distributional separation between patients with grade ≥ III complications and those without complications, reflected by a Cliff’s δ of −0.81, indicates that the finding was not driven solely by a small number of extreme observations. After covariate adjustment, grade ≥ III complications remained associated with four fewer median DAOH30 days—more than 13% of the entire observation window. Conversely, the adjusted estimate for grade I–II complications was smaller and its bootstrap confidence interval included zero. Thus, the magnitude and precision of the estimates indicate that the clearest separation was observed for complications requiring invasive intervention or higher-intensity management. Because complication severity directly influences hospitalization duration, the observed gradient represents an expected mechanistic association between postoperative morbidity and DAOH30. It should not be interpreted as evidence that DAOH30 provides incremental information beyond the index length of stay or the other components from which it is calculated.

This distinction is relevant because postoperative complications after gastrectomy are not simply binary events. Their severity determines the duration of hospitalization, the need for drainage or reoperation, nutritional interruption, additional imaging, antimicrobial treatment, and delayed functional recovery. Contemporary gastrectomy series have repeatedly demonstrated that major complications prolong recovery and are associated with adverse longer-term outcomes [14]. Large recent analyses have similarly shown that postoperative morbidity prolongs multiple components of recovery after radical gastrectomy and may adversely affect survival [25]. DAOH30 translates the downstream hospital-time consequences of postoperative events into a common unit—days lost from being alive outside the hospital. Complications with more prolonged or intensive consequences therefore tend to reduce DAOH30 more than events requiring brief treatment, which likely explains why the ordered severity analysis was more informative than a simple binary classification.

The hospital-time consequences of a major complication may depend not only on its Clavien–Dindo grade but also on its phenotype and management. This is particularly relevant for upper gastrointestinal anastomotic leaks, for which contemporary treatment may include percutaneous drainage, reoperation, covered stenting, endoscopic vacuum therapy, internal drainage, or endoscopic defect-closure techniques within a multidisciplinary strategy [26,27]. These approaches may alter the duration of hospitalization, the need for additional intervention, and the risk of readmission, all of which are reflected in DAOH30. Because only five anastomotic leaks occurred, the present study could not compare management strategies, and leak-specific findings remain descriptive.

The surgery-anchored 30-day readmission rate was 6.5%, but the eight events generated 73 additional inpatient days and a median DAOH30 decrement of 9.5 days per readmitted patient. This illustrates the descriptive advantage of quantifying readmission duration rather than reporting only a binary rate. Nevertheless, with only eight events, causes and subgroups should be interpreted descriptively. Readmission was treated as a component decomposition rather than an explanatory predictor because readmission days are algebraically embedded in DAOH30. Differences from prior gastrectomy series may also reflect surgery- versus discharge-anchored windows and variation in discharge and readmission pathways [10,11,15].

The index-cancer-excluded CCI analysis was exploratory and should be interpreted in light of restricted exposure variation: 77.3% of patients had scores of 0 or 1, only 8.9% had scores ≥ 3, and no early deaths occurred. No statistically significant association was detected between the index-cancer-excluded CCI and DAOH30; however, this finding should not be interpreted as evidence that baseline comorbidity is unimportant. Direct comparison with original or age-adjusted CCI studies also requires caution because the uniform weight assigned to the index malignancy was omitted and the descriptive categories used here are not externally validated risk strata. Frailty, nutritional status, sarcopenia, body composition, mobility, functional status, social support, and patient-reported recovery may be more informative for time spent alive outside the hospital and should be incorporated into future prospective studies [13,28,29].

At present, DAOH30 is best regarded as a complementary cohort-level measure for audits and perioperative trials, not a substitute for complication grading or length of stay and not a validated tool for individual counseling. Decomposition into index and readmission days may identify where hospital burden accumulates and can inform discharge education, nutritional surveillance, early symptom pathways, and rapid-access review. Future studies should include open, laparoscopic, and robotic practice across centers with different enhanced-recovery, intensive-care, discharge, and readmission pathways; evaluate DAOH30 and DAOH90 in parallel; and examine associations with quality of life, functional recovery, nutritional recovery, return to usual activity, and readiness for adjuvant treatment [30,31].

Strengths include a clinically homogeneous Surgical Oncology cohort, an explicitly defined 30-day window, source-verified hospitalization dates, continuous analysis without a data-driven threshold, and use of median differences, bootstrap confidence intervals, and distributional effect sizes. Readmission was analyzed as a component of DAOH30 rather than circularly entered as a predictor, quantile regression was used for the asymmetric outcome, and automated variable selection was avoided.

Several limitations should be acknowledged. First, the retrospective single-center design and complete-case eligibility introduce selection bias. In addition, admissions outside the institutional network could be captured only when documented during Surgical Oncology follow-up; any missed external readmission would have resulted in overestimation of DAOH30. Second, 118 of 123 procedures were open, and DAOH30 is sensitive to local intensive-care, nutritional, enhanced-recovery, discharge, and readmission pathways; the observed median of 21 days should therefore not be interpreted as a benchmark for gastrectomy or generalized to centers with predominantly laparoscopic or robotic practice. Third, only eight readmissions and five anastomotic leaks occurred, and no 30-day deaths were observed. Readmission and leak analyses are therefore descriptive, and the mortality-sensitive component of DAOH30 was not tested. Accordingly, these findings may not generalize to emergency surgery or to older, frailer, or higher-mortality populations in whom the mortality component of DAOH30 may contribute more substantially. Fourth, index length of stay is an algebraic component of DAOH30, so the strong inverse correlation was structural; the study does not establish incremental predictive value, net clinical benefit, or superiority over length of stay. Given the modest cohort size and the number of covariates included, adjusted regression estimates should be interpreted as explanatory and may remain imprecise despite the consistency of the sensitivity analyses. Fifth, the index-cancer-excluded CCI was retrospectively derived, had a restricted distribution, and is not interchangeable with the original or age-adjusted CCI. Finally, DAOH30 does not directly measure functional recovery, quality of life, patient preference, outpatient treatment burden, or readiness for adjuvant therapy, and the study did not establish convergent validity, responsiveness, or a minimal clinically important difference.

## 5. Conclusions

DAOH30 summarized realized early hospital burden after R0 total gastrectomy or subtotal distal gastrectomy for gastric adenocarcinoma. The observed complication-severity gradient reflects the expected mechanistic relationship between postoperative morbidity, prolonged hospitalization, and DAOH30, whereas eight readmissions contributed 73 additional inpatient days. Because no 30-day deaths occurred and most patients were not readmitted, DAOH30 was largely determined by index length of stay and should be considered a complementary descriptive measure rather than evidence of incremental value beyond conventional postoperative outcomes. Multicenter prospective validation across diverse surgical and perioperative pathways, with patient-reported anchoring, is required.

## Figures and Tables

**Table 1:** Clinical characteristics and early postoperative outcomes of the primary cohort.

Variable	Overall Cohort (*n* = 123)
**Patient Characteristics**	
Age, years	67 (57–75)
Male sex	73 (59.3%)
ASA class III–IV	67 (54.5%)
ECOG performance status ≥ 1	91 (74.0%)
Neoadjuvant treatment	22 (17.9%)
**Operative Characteristics**	
Subtotal distal gastrectomy	57 (46.3%)
Total gastrectomy	66 (53.7%)
Open surgical approach	118 (95.9%)
**Comorbidity Burden**	
Index-cancer-excluded CCI, median (IQR)	1 (0–1)
Score 0	50 (40.7%)
Score 1	45 (36.6%)
Score 2	17 (13.8%)
Score ≥ 3	11 (8.9%)
Age-adjusted index-cancer-excluded CCI, median (IQR)	3 (2–4)
**Early Postoperative Outcomes**	
Index postoperative length of stay, days	9 (8–11)
No postoperative complication	47 (38.2%)
Clavien–Dindo grade I–II	49 (39.8%)
Clavien–Dindo grade ≥ III	27 (22.0%)
Anastomotic leakage	5 (4.1%)
Reoperation within 30 days	9 (7.3%)
Readmission within 30 days	8 (6.5%)
30-day mortality	0 (0.0%)
DAOH30, median (IQR), days	21 (18–22)
DAOH30, mean ± SD, days	20.0 ± 4.2

Data are presented as median (interquartile range), *n* (%), or mean ± standard deviation, as appropriate. CCI values refer to the index-cancer-excluded CCI unless otherwise specified. ASA, American Society of Anesthesiologists; CCI, Charlson Comorbidity Index; DAOH30, days alive and out of hospital within 30 days after surgery; ECOG, Eastern Cooperative Oncology Group; IQR, interquartile range; SD, standard deviation.

**Table 2:** Known-groups discrimination of DAOH30. (**A**) DAOH30 across increasing postoperative complication severity. (**B**) Supportive clinical comparisons.

(A)							
Postoperative Complication-Severity Group			*n*	DAOH30, Median (IQR), Days			
No postoperative complication			47	22 (21–23.5)			
Clavien–Dindo grade I–II			49	21 (18–22)			
Clavien–Dindo grade ≥ III			27	17 (12.5–19.5)			
**Overall comparison**			123	**Kruskal–Wallis *p* < 0.001**			
**Ordered severity trend**			123	**One-sided Jonckheere–Terpstra *p* < 0.001**			
Two-sided sensitivity analysis			123	Permutation *p* < 0.001			
(**B**)							
**Comparison**	***n*, First-Listed/Reference**	**DAOH30 in First-Listed Group, Median (IQR), Days**	**DAOH30 in Reference Group, Median (IQR), Days**		**Median Difference, Days**	**Bootstrap 95% CI**	**Unadjusted *p* Value**
Major complication versus no major complication	27/96	17 (12.5–19.5)	21 (20–23)		−4	−6 to −2	<0.001
Reoperation within 30 days versus no reoperation	9/114	18 (17–19)	21 (19–22)		−3	−5 to 0	0.009
Anastomotic leakage versus no leakage	5/118	18 (10–21)	21 (19–22)		−3	−13 to 2	0.170
Total versus subtotal distal gastrectomy	66/57	20 (18–22)	21 (19–22)		−1	−2 to 0	0.185

Median differences were calculated as the first-listed group minus the reference group; negative values indicate fewer DAOH30 days in the first-listed group. Median-difference confidence intervals were obtained using 100,000 nonparametric bootstrap resamples. (**B**) comparisons were exploratory; *p* values were derived from Mann–Whitney U tests and were not adjusted for multiplicity. Mann–Whitney *p* values assess differences in rank distributions, whereas bootstrap confidence intervals quantify median differences; because these procedures address different estimands, their inferential conclusions need not coincide. Detailed Dunn tests with Holm adjustment and Cliff’s δ estimates for the ordered complication-severity comparisons are provided in Supplementary Table S2. CI, confidence interval; DAOH30, days alive and out of hospital within 30 days; IQR, interquartile range. The two-sided sensitivity *p* value was based on the absolute deviation of the observed Jonckheere–Terpstra statistic from its permutation null expectation using 50,000 label permutations.

**Table 3:** Adjusted median quantile regression models for DAOH30. (**A**) Preoperative Charlson association model. (**B**) Adjusted complication-severity model.

(A)			
Covariate	Adjusted Median Difference in DAOH30, Days	95% Percentile-Bootstrap CI	Model-Based *p* Value
Index-cancer-excluded CCI, per 1-point increase	−0.17	−1.34 to 0.40	0.640
Age, per 10-year increase	0.20	−0.68 to 0.94	0.551
Male sex	0.91	−0.75 to 2.50	0.222
ASA class III–IV versus I–II	−0.41	−2.11 to 0.95	0.593
Total versus subtotal distal gastrectomy	−0.52	−2.18 to 0.76	0.472
Neoadjuvant treatment	−0.37	−3.00 to 1.89	0.696
Calendar year of surgery	0.07	−0.55 to 0.52	0.820
(**B**)			
**Covariate**	**Adjusted Median Difference in DAOH30, Days**	**95% Percentile-Bootstrap CI**	**Model-Based *p* Value**
Clavien–Dindo grade I–II versus no postoperative complication	−1.00	−3.00 to 0.00	0.180
Clavien–Dindo grade ≥ III versus no postoperative complication	−4.00	−7.31 to −2.25	<0.001
Index-cancer-excluded CCI, per 1-point increase	0.00	−1.12 to 0.46	>0.99
Age, per 10-year increase	0.00	−0.69 to 0.97	>0.99
Male sex	1.00	−0.79 to 2.31	0.142
ASA class III–IV versus I–II	0.00	−1.27 to 1.42	>0.99
Total versus subtotal distal gastrectomy	0.00	−1.85 to 0.95	>0.99

(**A**) included the index-cancer-excluded CCI, age, sex, ASA class, gastrectomy type, neoadjuvant treatment, and calendar year of surgery. (**B**) included complication-severity group, the index-cancer-excluded CCI, age, sex, ASA class, and gastrectomy type. Coefficients represent adjusted differences in median DAOH30; negative coefficients indicate fewer days alive and out of hospital. Ninety-five percent percentile-bootstrap confidence intervals were obtained using 5000 nonparametric case-resampling samples; *p* values were obtained from the fitted median quantile regression models. The maximum variance inflation factor in the preoperative Charlson association model was 1.27. ASA, American Society of Anesthesiologists; CCI, Charlson Comorbidity Index; CI, confidence interval; DAOH30, days alive and out of hospital within 30 days. No statistically significant association between the index-cancer-excluded CCI and DAOH30 was detected in any sensitivity analysis; all corresponding 95% confidence intervals included zero (Supplementary Table S4).

## Data Availability

The aggregate data supporting the findings of this study are presented in the article and its Supplementary Materials. The de-identified patient-level data may be made available by the corresponding author upon reasonable request, subject to approval by Ankara Bilkent City Hospital and compliance with the ethics approval and applicable institutional data-protection requirements. The data are not publicly available because they contain sensitive clinical information and unrestricted sharing could compromise patient confidentiality.

## Supplementary Materials

The following supporting information can be downloaded at: https://www.mdpi.com/article/10.3390/jcm15176811/s1, Table S1: Prevalence of Components Included in the Index-Cancer-Excluded Charlson Comorbidity Index; Table S2: Pairwise Comparisons Across Postoperative Complication-Severity Groups; Table S3: Clinical and Temporal Characteristics of 30-Day Readmission Episodes; Table S4: Sensitivity Analyses of the Association Between the Index-Cancer-Excluded CCI and DAOH30; Table S5: DAOH30 According to Index-Cancer-Excluded CCI Category.
