# Supplementary material for: Days Alive and out of Hospital at 30 Days After Curative Gastrectomy for Gastric Adenocarcinoma: Complication-Severity Gradient and Readmission Burden
Source: J Clin Med. 2026 Sep 2;15(17):6811. doi: 10.3390/jcm15176811 (PMC13566587; doi:10.3390/jcm15176811)
Supplement: Supplementary file 1 [file jcm-15-06811-s001.zip › supplementary Table S1.pdf]

Supplementary Table S1. Prevalence of Components Included in the Index-Cancer-Excluded Charlson Comorbidity Index

Charlson Component	n	%
Diabetes without chronic complications	21	17.1
Myocardial infarction	17	13.8
Chronic pulmonary disease	15	12.2
Congestive heart failure	10	8.1
Moderate-to-severe renal disease	8	6.5
Cerebrovascular disease	7	5.7
Peptic-ulcer disease	7	5.7
Peripheral vascular disease	6	4.9
Connective-tissue disease	4	3.3
Mild liver disease	4	3.3
Lymphoma	3	2.4
Diabetes with chronic complications	2	1.6
Dementia	1	0.8
Hemiplegia or paraplegia	0	0.0
Leukemia	0	0.0
Moderate-to-severe liver disease	0	0.0
Metastatic solid tumor	0	0.0
AIDS/HIV	0	0.0

Comorbidities were determined from source-verified preoperative documentation. Mutually exclusive severity categories were not scored simultaneously. The uniform weight assigned to the index non-metastatic gastric malignancy was omitted from the index-cancer-excluded CCI.
