# Supplementary material for: Days Alive and out of Hospital at 30 Days After Curative Gastrectomy for Gastric Adenocarcinoma: Complication-Severity Gradient and Readmission Burden
Source: J Clin Med. 2026 Sep 2;15(17):6811. doi: 10.3390/jcm15176811 (PMC13566587; doi:10.3390/jcm15176811)
Supplement: Supplementary file 1 [file jcm-15-06811-s001.zip › supplementary Table S2.pdf]

Supplementary Table S2. Pairwise Comparisons Across Postoperative Complication-Severity Groups

Pairwise Comparison	Median Difference, Days	Bootstrap 95% CI	Dunn z	Holm-Adjusted p Value	Cliff's δ
Clavien–Dindo grade I–II versus no postoperative complication	−1	−3 to 0	−2.815	0.005	−0.347
Clavien–Dindo grade ≥ III versus no postoperative complication	−5	−6 to −3	−6.029	< 0.001	−0.809
Clavien–Dindo grade ≥ III versus grade I–II	−4	−5 to −1	−3.676	< 0.001	−0.536

Median differences were calculated as the first-listed severity group minus the reference group. Pairwise rank comparisons were performed using Dunn tests with Holm adjustment. Negative Cliff's δ values indicate lower DAOH30 in the first-listed group. Dunn–Holm p values assess differences in rank distributions, whereas bootstrap confidence intervals quantify median differences; because these procedures address different estimands, their inferential conclusions need not coincide.
