# Supplementary material for: Days Alive and out of Hospital at 30 Days After Curative Gastrectomy for Gastric Adenocarcinoma: Complication-Severity Gradient and Readmission Burden
Source: J Clin Med. 2026 Sep 2;15(17):6811. doi: 10.3390/jcm15176811 (PMC13566587; doi:10.3390/jcm15176811)
Supplement: Supplementary file 1 [file jcm-15-06811-s001.zip › supplementary Table S3.pdf]

Supplementary Table S3. Clinical and Temporal Characteristics of 30-Day Readmission Episodes

Episode	Readmission POD	Index LOS, Days	Readmission Days Within 30-Day Window	DAOH30	Clavien–Dindo Grade	Principal Reason for Readmission
A	17	14	12	4	IIIa	Intra-abdominal abscess/collection
B	19	15	11	4	IIIb	Intra-abdominal abscess/collection
C	15	12	11	7	IIIa	Intra-abdominal abscess/collection
D	20	16	6	8	IIIa	Abdominal sepsis/intra-abdominal abscess
E	12	9	12	9	II	Nutrition/dehydration or medical cause
F	13	10	8	12	II	Nutrition/dehydration or medical cause
G	10	7	8	15	II	Infection/fever
H	12	6	5	19	II	Infection/fever

Episodes are ordered by ascending DAOH30 to match Figure 4. Readmission POD was calculated from the operation date. Only inpatient days overlapping the first 30 postoperative days contributed to DAOH30. DAOH30, days alive and out of hospital within 30 days; LOS, length of stay; POD, postoperative day.
