# Supplementary material for: Days Alive and out of Hospital at 30 Days After Curative Gastrectomy for Gastric Adenocarcinoma: Complication-Severity Gradient and Readmission Burden
Source: J Clin Med. 2026 Sep 2;15(17):6811. doi: 10.3390/jcm15176811 (PMC13566587; doi:10.3390/jcm15176811)
Supplement: Supplementary file 1 [file jcm-15-06811-s001.zip › supplementary Table S4.pdf]

Supplementary Table S4. Sensitivity Analyses of the Association Between the Index-Cancer-Excluded CCI and DAOH30

Sensitivity Analysis	Exposure	n	Adjusted Median Difference, Days	95% CI	Model-Based p Value	CI Method
Preoperative Charlson association model	Index-cancer-excluded CCI per 1-point increase	123	−0.17	−1.34 to 0.40	0.640	5000-resample percentile bootstrap
Age-adjusted score substituted for separate score and age terms	Age-adjusted index-cancer-excluded CCI per 1-point increase	123	0.00	−0.44 to 0.44	> 0.99	Model-based
Index-cancer-excluded CCI categorical model	Score 1 versus 0	123	−0.65	−2.28 to 0.99	0.435	Model-based
Index-cancer-excluded CCI categorical model	Score 2 versus 0	123	0.09	−2.17 to 2.35	0.938	Model-based
Index-cancer-excluded CCI categorical model	Score ≥ 3 versus 0	123	−1.97	−4.63 to 0.69	0.145	Model-based
ECOG substituted for ASA	Index-cancer-excluded CCI per 1-point increase	123	−0.33	−1.05 to 0.38	0.358	Model-based
Open gastrectomy only	Index-cancer-excluded CCI per 1-point increase	118	−0.37	−1.05 to 0.31	0.289	Model-based

Negative coefficients indicate fewer median DAOH30 days. In the age-adjusted score model, chronological age was not entered separately. The primary-model confidence interval was obtained using 5000 nonparametric case-resampling bootstrap samples; confidence intervals for sensitivity models were model-based. All reported p values were model-based. ASA, American Society of Anesthesiologists; CCI, Charlson Comorbidity Index; CI, confidence interval; ECOG, Eastern Cooperative Oncology Group.
