# Supplementary material for: Days Alive and out of Hospital at 30 Days After Curative Gastrectomy for Gastric Adenocarcinoma: Complication-Severity Gradient and Readmission Burden
Source: J Clin Med. 2026 Sep 2;15(17):6811. doi: 10.3390/jcm15176811 (PMC13566587; doi:10.3390/jcm15176811)
Supplement: Supplementary file 1 [file jcm-15-06811-s001.zip › supplementary Table S5.pdf]

Supplementary Table S5. DAOH30 According to Index-Cancer-Excluded CCI Category

Index-Cancer-Excluded CCI Category	n	DAOH30, Median (IQR), Days
Score 0	50	21 (19–23.8)
Score 1	45	21 (19–22)
Score 2	17	21 (20–22)
Score ≥ 3	11	19 (17.5–21.5)
Overall comparison	123	Kruskal–Wallis $p = 0.391$
Ordinal association	123	Kendall's $\tau b = -0.108$; $p = 0.136$

The index-cancer-excluded CCI was calculated without chronological age and without adding the uniform weight assigned to the index non-metastatic gastric malignancy. CCI, Charlson Comorbidity Index; DAOH30, days alive and out of hospital within 30 days; IQR, interquartile range.
